# In Vivo and In Vitro Assessment and Proteomic Analysis of the Effectiveness of Physical Treatments in Reducing Allergenicity of Hazelnut Proteins

**DOI:** 10.3390/nu14040874

**Published:** 2022-02-19

**Authors:** Elisabetta De Angelis, Danilo Di Bona, Rosa Pilolli, Roberto Loiodice, Anna Luparelli, Lucia Giliberti, Angela M. D’Uggento, Maria Pia Rossi, Luigi Macchia, Linda Monaci

**Affiliations:** 1 Institute of Sciences of Food Production—National Research Council of Italy (ISPA-CNR), 70126 Bari, Italy; elisabetta.deangelis@ispa.cnr.it (E.D.A.); rosa.pilolli@ispa.cnr.it (R.P.); anna.luparelli@uniba.it (A.L.); 2 Department of Emergency and Organ Transplantation (DETO), School and Chair of Allergology and Clinical Immunology, University of Bari Aldo Moro, 70124 Bari, Italy; danilo.dibona@uniba.it (D.D.B.); robertoloiodice@tiscali.it (R.L.); lucia.giliberti@uniba.it (L.G.); mariapia.rossi@uniba.it (M.P.R.); luigi.macchia@uniba.it (L.M.); 3 Department of Economics and Finance, University of Bari Aldo Moro, 70124 Bari, Italy; angelamaria.duggento@uniba.it

**Keywords:** food allergy, allergens, allergenicity reduction, skin prick test, proteomic analysis, physical treatments

## Abstract

Hazelnut is a widespread nut species, especially present in Europe, that can be consumed raw or roasted thanks to its pleasant taste and nutritional properties. In addition to renowned beneficial properties hazelnuts contain several proteins capable of inducing food allergy in sensitized individuals, including Cor a 2 (a profilin), Cor a 8 (a lipid transfer protein), Cor a 9 (an 11S seed storage globulin, legumin-like), and Cor a 11 (a 7S seed storage globulin, vicilin-like). In the present paper we investigated the effectiveness of autoclave-based treatments in decreasing the allergic potential of hazelnut as assessed by submitting the treated material to an in vivo skin prick test and an in vitro immunoblot analysis, with sera of allergic individuals exposed to the treated food material. This preliminary analysis showed that autoclave treatment preceded by hydration and/or followed by drying seems to be a promising approach and appears to be effective in reducing the allergenicity of hazelnuts in most patients, probably due to the denaturation of most major and minor allergenic proteins. This work opens up the opportunity to produce hypoallergenic hazelnut derivatives that can be tolerated by allergic subjects.

## 1. Introduction

The hazelnut is one of the foods most frequently responsible for allergic reactions, with an estimated prevalence in Europe of approximately 9.3% of hazelnut specific IgE detected in adults (20–54 years) [1,2,3]. Several allergens have been identified in hazelnuts and are included in the WHO-IUIS list of allergens [4], with the most-studied proteins in relation to food allergy being Cor a 2 (profilin), Cor a 8 (lipid transfer protein), Cor a 9 (11S seed storage globulin, legumin-like) and Cor a 11 (7S seed storage globulin, vicilin-like). Sensitization to Cor a 2 (thermo- and gastro-labile) is associated with oral allergic syndrome (OAS), whereas sensitization to Cor a 8, 9 or 11 (thermo- and gastro-resistant) is generally associated with systemic and even severe and life-threatening reactions. Hazelnut consumption is widespread all over the world, especially in Europe and in the Mediterranean diet, due to the nut’s pleasant taste and nutritional properties. Hazelnuts can be consumed fresh or roasted, and are often used as an ingredient in food products or preparations such as spreads, baked goods, pastry, chocolates, and confectionary products. Although common preparation processes can modify food proteins, they do not necessarily reduce the allergenic potential of the food in question, especially in the case of thermo-resistant proteins, such as is the case for nut proteins belonging to the class of seed storage globulins, which maintain their allergenicity even after roasting. European regulation 1169/2011 on the labeling of food products mandates obligatory and clear labeling for fourteen allergenic ingredients, including nuts [5]. On the other hand, hazelnut proteins can contaminate food products as a “hidden allergen” as a result of cross-contamination occurring during manufacturing; this can inadvertently expose allergic consumers to its ingestion. The identification of physical treatments capable of reducing the potential of hazelnut allergens to trigger reactions in sensitized individuals can pave the way for the production of hypoallergenic hazelnut derivatives tolerated by allergic subjects; this kind of investigation has already been carried out by our team for almonds, with an evaluation done in vitro [6].

In the present study, we investigated the effects on hazelnuts subjected to autoclaving under different schemes, preceded or not by hydration and eventually followed by drying; after obtaining treated hazelnut extracts, we assessed their protein composition, performing gel electrophoresis, western blot and mass spectrometry, and testing their skin reactivity by performing skin prick testing in patients with hazelnut allergy.

## 2. Materials and Methods

### 2.1. Patient Enrollment in the Study

Twenty-two patients with presumed hazelnut allergy were selected at the University Hospital of Bari (Unit of Allergology). Presumed hazelnut allergy (no oral food challenge was performed to confirm diagnosis) was defined based on such allergic symptoms following hazelnut ingestion as urticaria, angioedema, oral allergy syndrome, asthmatic symptoms, anaphylaxis followed by a positive skin prick test [7].

Skin prick tests (SPTs) were carried out on the volar surface of the forearm by puncturing the skin with a 1 mm point length standardized needle (ALK-Abellò, Milan, Italy) to admit a 100 µg/mL droplet of the allergen. Histamine 10 mg/mL was used as the positive control. SPTs were performed by placing a drop of extract on the skin of the forearm and subsequently pricking the skin underneath this drop. The extracts of the different treated hazelnut samples (A1, A2, A3, A4) were tested on the patients simultaneously. The appearance of a wheal with a mean diameter of at least 3 mm was considered a positive response. Skin reactivity was expressed by measuring the area of the wheals (mm^2^). All SPTs were performed in duplicate for each allergen.

Patients underwent skin testing with three different commercial extracts of hazelnut (Lofarma^®^ (Milan, Italy) 2% *w*/*v*, ALK-Abello^®^ (Horsholm, Denmark)1:20 *w*/*v*, and Alyostal^®^ (Stallergenes^®^, London UK) 1 IC/mL) and the specifically prepared raw hazelnut seed extracts (*Corylus avellana*, var. Italiana) at a concentration of 2 mg/mL, left untreated (unmodified, denominated A1), or subjected to differential physical treatments (marked as A2, A3 and A4, as detailed below). 

Saline was used as the negative control for the commercial extracts, while a urea buffer solution used as a diluent for all of the hazelnut extracts was used as the negative control for the hazelnut extracts.

Sera of 14 of the 22 included patients were obtained and used for subsequent immunoblot experiments.

### 2.2. Chemicals

The study was performed on raw hazelnut seeds (*Corylus avellana*, var. Italiana) provided by Besana S.p.A. (San Gennaro Vesuviano, Naples, Italy). Trizma-base, urea, sodium chloride, ammonium bicarbonate (AMBIC), iodoacetamide (IAA), dithiothreitol (DTT), and all chemicals for electrophoresis, namely sodium dodecyl sulfate-SDS, glycine, glycerol, Coomassie brilliant blue-G 250, were purchased by Sigma Aldrich (Milan, Italy). Acetonitrile (ACN, Gold HPLC ultragradient), Methanol (HPLC grade), trifluoroacetic acid (TFA) and Bromophenol blue were obtained from Carlo Erba Reagents (Cornaredo, Milan, Italy). Ultrapure water was produced by a Millipore Milli-Q system (Millipore, Bedford, MA, USA), while formic acid (FA, MS grade) was purchased from Fluka (Milan, Italy). Polytetrafluoroethylene (PTFE) filters (0.45µm) were obtained from Sartorius (Gottingem, Germania), and syringe filters in cellulose acetate (CA) 1.2 µm were purchased from Labochem Science S.r.l. (Catania, Italy). In-gel protein digestion experiments were accomplished using trypsin (proteomic grade) obtained from Promega (Milan, Italy).

### 2.3. Hazelnut Autoclave Treatments

Autoclaving treatments were accomplished on a total of three raw hazelnut kernels (corresponding to approximately 15 g) placed into a centrifuge tube. Specifically, three different processing schemes were taken into consideration: (i) autoclaving, (ii) sample pre-hydration followed by autoclaving, and (iii) sample pre-hydration followed by autoclaving and finally drying in a stove overnight at 60 °C. For the hydration step, raw hazelnut kernels were soaked in 50 mL of ultrapure water and left shaking for 2 h at room temperature in an orbital shaker (KS 4000 i-control shaker, IKA Works GmbH & Co. KG, Staufen, Germany). Water was discarded before autoclaving. Autoclaving was accomplished by setting the equipment with a temperature of 134 °C, pressure of 2 atm, and cycle time of 10 min. Autoclaving treatment lasted approximately 1 h, of which 40 min was taken to reach the temperature of 134 °C.

The three different schemes investigated can be summarized as:

(a) Hazelnut autoclaved for 10 min (A2)
(b) Hazelnut prehydrated + autoclaved (A3)
(c) Hazelnut prehydrated + autoclaved + drying (A4).

Raw hazelnut not submitted to any treatment (A1) was used as a positive control.

**Figure 1:**
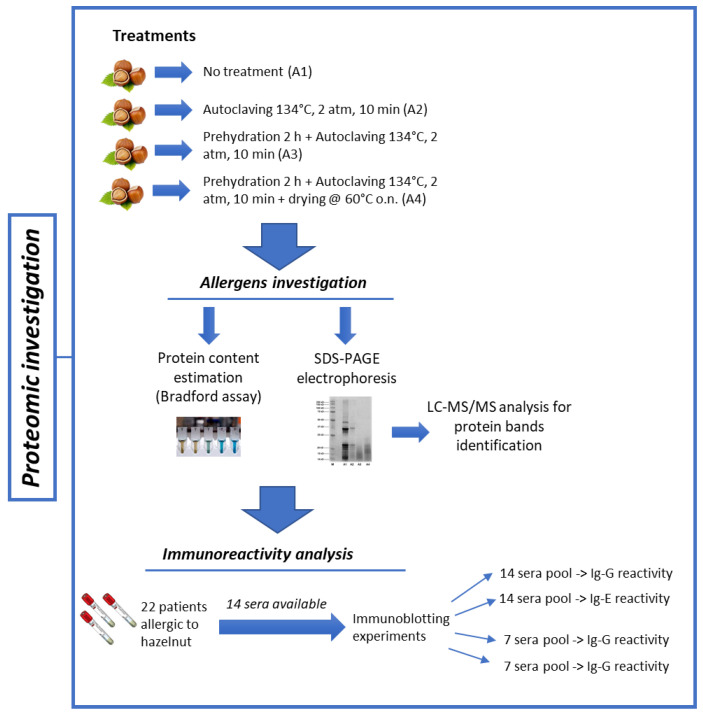
Workflow for proteomic investigation, encompassing the extraction step, electrophoretic analysis, protein identification by Mass Spectrometry, and immunoblot analysis for immunoreactivity assessment. Sera were available for 14 of 22 patients.

The workflow of the proteomic analysis carried out in this work is reported in Figure 1.

### 2.4. Protein Extraction and Quantification

Before analysis, raw and treated hazelnut seeds were milled using an electric miller (Mulinex, Milan, Italy) and proteins were extracted by following the protocol described by Bavaro et al., 2018 [8]. The samples were filtered through 1.2 µm CA syringe filters before successive analysis. 

A ready-to-use colorimetric Bradford assay (Quick Start™ Bradford Protein Assay, Bio-Rad Laboratories s.r.l., Segrate MI, Italy) was used to estimate the total protein content of the raw and autoclaved hazelnut. The analysis was accomplished following the manufacturer’s instruction with bovine serum albumin (BSA, 0.125–1 mg/mL) protein used as the reference standard. Samples were stored at −20 °C for further analysis and filtered through 0.45 µm PTFE filters immediately prior to electrophoretic runs.

### 2.5. Electrophoretic Analysis of Hazelnut Proteins

Proteins extracted from raw and processed hazelnut samples were profiled by means of sodium dodecyl sulphate–polyacrylamide gel electrophoresis (SDS-PAGE). Specifically, ten micrograms of proteins were separated under reducing condition on an 8–16% polyacrylamide pre-cast gel (13.3 cm × 8.7 cm × 1.0 mm) using Mini-Protean Tetra Cell equipment (Bio-rad Laboratories, Segrate, MI, Italy). Prior to electrophoresis analysis, samples were denatured with Laemmli buffer (62.5 mM TrisHCl, pH 6.8, 25% glycerol, 2% SDS, 0.01% Bromophenol Blue, 100 mM DTT) at a 1:1 ratio for 5 min at 100 °C. Electrophoretic runs were accomplished according to the following conditions: 60 V for the first 20 min, then 100 V until the end of the run, using a Tris Glycine SDS (TGS, 25 mM Tris, 192 mM glycine, 0.1% SDS) solution as running buffer. Gels were stained with Coomassie Brilliant Blue G-250 solution and the protein profiles detected on a ChemiDoc™ Imaging System (Bio-Rad Laboratories, Segrate, MI, Italy). Precision Plus Protein™ all blue standard (10–250 kDa, Bio-Rad Laboratories, Segrate, MI, Italy) was used as a protein reference for molecular weight.

### 2.6. In-Gel Tryptic Digestion and LC-MS Analysis

The electrophoretic profiles of raw and treated hazelnut samples were then subjected to deep inspection in order to find the most informative protein bands to be submitted to in-gel digestion for identification purposes. Briefly, the selected bands were excised from the polyacrylamide gels and in-gel trypsin digested according to the protocol described by De Angelis et al., 2017 [9]. After drying, each sample was re-suspended in 100 µL of H_2_O/ACN 95/5 + 0.1% formic acid (*v*/*v*) and 5 µL were injected into LC/MS apparatus.

Peptide mixtures obtained from protein bands in-gel digested and referred to samples A1, A2, A3 and A4 were analyzed by untargeted proteomic analysis using HPLC-MS/MS equipment consisting of a Q-Exactive™ Plus Hybrid Quadrupole-Orbitrap™ Mass Spectrometer coupled to a UHPLC pump system (Thermo Fisher Scientific, Bremen, Germany). For the peptide chromatographic separation, a reversed phase Acclaim™ PepMap100 C18 analytical column (1 mm × 15 cm × 3 m, 100 Å porosity, Thermo Fisher Scientific, Bremen, Germany) was used, with the flow rate set at 60 µL/mL. The elution gradient was as follows: an increase of solvent B from 10% to 60% in 60 min, then a further increase from 60% to 80% in 1 min. These conditions were kept constant for 10 min and then returned to initial values for column conditioning before the next injection (20 min conditioning); solvent A = H_2_O + 0.1% FA, and solvent B = ACN/H_2_O (80/20 *v*/*v*) + 0.1% FA. Volume injection was set to 5 µL, and each sample was injected twice in MS. Spectra were acquired in the mass range of 150–2000 m/z and the data-dependent acquisition mode (FullMS-dd2) was used for MS runs, taking into account only positive ions. The method parameters and HESI source conditions were the same as previously described by Bavaro et al. 2018 [8], with the exceptions of normalized collision energy (NCE) value, which was set at 27 and 30 eV by activating the stepped option, and automatic gain control (AGC) target, which was set to 1.00 × 10^6^ for the MS^1^ event and to 5.00, with an intensity threshold of 1.0 × 10^3^, for MS^2^ analysis.

MS spectra were then processed via the commercial software Proteome Discoverer™ version 2.1.1.21 (Thermo-Fisher-Scientific, Bremen, Germany), which is based on SequestHT algorithms for protein identification. A search was carried out against the hazelnut protein database extracted by Swiss ProtDB on the basis of the taxonomy code of *Corylus avellana* (ID: 13451, containing about 501 sequences), selecting trypsin as the cleavage enzyme. Protein identification was accomplished according to the criteria described by Bavaro et al. 2018 [8], taking into account peptide sequences assigned with at least medium confidence (FDR < 5%).

### 2.7. Immunoblot for IgE-Binding Assay

Proteins of the untreated and different processed hazelnuts were separated by SDS-PAGE (corresponding to 10 µg of proteins loaded for A1, A2, A3 and A4) and electroblotted onto 0.2 µm nitrocellulose membranes (Bio-Rad Laboratories, Segrate, MI, Italy) using Trans-Blot Cell (Bio-Rad Laboratories, Segrate, MI, Italy) for 7 min (1.3 A, 25 V). Immunoblotting analyses were accomplished as described by Bavaro et al., 2019 [10]. Among the 22 patients recruited for the study, only 14 gave their consent to blood sampling for further investigation; therefore, immunoblotting analyses were accomplished only on these 14 allergic individuals, who showed various clinical symptoms (urticaria, itching, hands/throat angioedema). In detail, the pooled serum of the 14 individuals was diluted in TBS-T (pH 7.4, 10 mM Tris, 50 mM NaCl, 0.1% Tween 20) at a 1:50 ratio and used as a primary antibody. Electroblotted membrane was incubated with the pooled serum overnight at 4 °C in a tilting agitator gently shaking. As secondary antibodies, goat anti-human IgG (H + L) horseradish peroxidase (HRP) conjugated (Bio-Rad Laboratories) and anti-human IgE (ε-chain specific) horseradish peroxidase conjugated (Sigma Aldrich, Milan Italy) each diluted 1/5000 (*v*/*v*) in TBS-T were added. Specifically, four different immunoblotting experiments were accomplished; in the first two experiments, two different serum pools (each obtained by combining the sera of seven patients per group) were used, with the reactive bands detected using the goat anti-human IgG (H + L) horseradish peroxidase (HRP) conjugate (vide infra) as the secondary antibody. In the third and fourth experiments the sera of all 14 patients were pooled together, and the reactive proteins were identified after incubation with anti-human IgE (ε-chain specific) peroxidase secondary antibody binding and goat anti-human IgG (H + L) horseradish peroxidase (HRP) (vide infra). Final images were obtained on a ChemiDoc™ MP Imaging System.

### 2.8. Statistical Analysis

All data were analysed using SPSS software, version 25.0.0 (IBM, Segrate, MI, Italy). Data were shown as median and interquartile range (IQR) if not normally distributed. To test the normality of distributions, the Shapiro–Wilk test was used. The Wilcoxon test for paired samples was used when investigating the possible differences between paired medians. Differences were considered significant at *p* < 0.05, and all values are two sided.

## 3. Results and Discussion

### 3.1. Effects of Hazelnut Thermal/Pressure Treatment on Patient Response

First, we sought to assess whether the modified hazelnut extracts were associated with a reduced patient response in vivo. To this end, 22 patients with diagnosed hazelnut allergy (16 females namely 72.7%; mean age, 28.1 ± 9.4 years; range: 17–48 years) underwent skin prick testing [11]. Specifically, 19 (86.4%) of 22 patients reported urticaria/angioedema, three reported oral allergy syndrome (13.6%), and one reported both manifestations (4.5%), suggesting that most patients were likely sensitized to hazelnut proteins such as Cor a 8, Cor a 9, Cor a 11 that are known to be responsible for systemic symptoms (Table 1).

The SPT reactivity (defined as the mean wheal area of two independent measurements) reported with the hazelnut extract A1 (unmodified hazelnut seed extracts-*Corylus avellana*, var. Italiana) was 24.6 ± 12.3 mm^2^, comparable to that produced by the three commercial extracts (ALK, 26.5 ± 13.2 mm^2^; Stallergenes, 22.8 ± 12.3 mm^2^; Lofarma, 18.7 ± 9.0 mm^2^). This suggested that the native A1 extract was effective and suitable for use for comparison with the modified extracts. Negative controls (either saline or urea solutions) did not elicit any skin reaction (no wheal, erythema, or pruritus).

**Figure 2:**
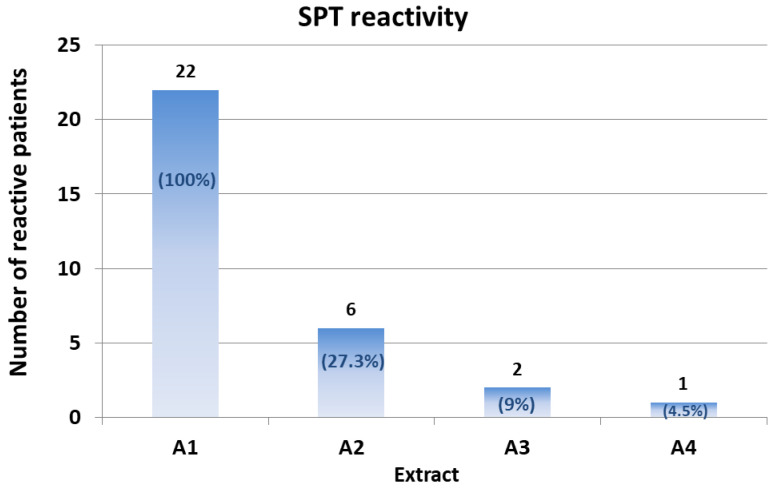
Number and percentage of SPT-positive patients to the different treated hazelnut extracts; the number and percentage (in brackets) of reactive patients are reported in each column.

Only six patients (27.3%) showed reactivity to the A2 extract. This reactivity appeared lower than that of the native extract (the mean wheal area of the six reactive patients was 15.2 ± 4.1 mm^2^; *p* for A1 vs. A2 = 0.005) (Figure 2; Table 2). 

Only two of the six patients reactive to A2 showed reactivity to the A3 extract. The mean wheal area of the two reactive patients was 13.5 mm^2^ (patient #15) and 4.5 mm^2^ (patient #20), respectively (Figure 2; Table 2).

Finally, only one patient (patient #15) showed SPT reactivity (wheal area, 13.5 mm^2^) to the A4 extract.

Collectively, these results suggest that all of the three different treatments are effective in reducing hazelnut allergenicity in a meaningful proportion of patients. Treatments 3 and 4 appeared to be the most effective. Notably, one patient out of 22 did not show a significant reduction in SPT reactivity even with the A4 extract. Further analyses remain necessary in order to define the specific epitopes this patient was reactive to.

### 3.2. Effect of Thermal/Pressure Treatment on Hazelnut Protein Solubility

**Figure 3:**
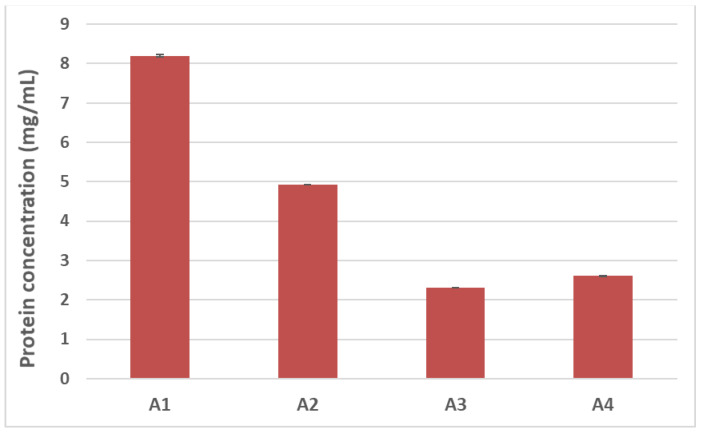
Protein content as estimated by Bradford protein assay, corresponding to untreated hazelnut extract (A1) and hazelnut extract submitted to autoclaving (A2), pre-hydration/autoclaving (A3), and pre-hydration/autoclaving followed by drying (A4).

Food processing is known to alter the final structure and function of proteins, modifying such crucial properties as final solubility. Denaturation, hydrolysis of peptide bonds, restructuring of disulphide bonds, and interaction with other components (i.e., carbohydrates and lipids) can frequently occur during treatment and are among the causes leading to reduced protein solubility [12]. In light of this, we first sought to evaluate how autoclaving/pressure processing could alter the final solubility of hazelnut proteins by estimating the total protein content of samples undergoing the different treatments. The Bradford assay was used for this purpose (Figure 3). A progressive reduction in protein recovery was shown in the treated hazelnut samples compared to the untreated counterpart. The total protein content of the autoclaved samples (A2) appeared to be 40% lower than the control sample (A1), with a more dramatic reduction observed for the pre-hydrated/autoclaved sample (A3) and the pre-hydrated/autoclaved/dried samples (A4), which showed a 70% decrease in protein recovery compared to the untreated hazelnut sample (Figure 3).

The results presented here are in accordance with previous studies reporting the effects of autoclaving on other nuts species such as almonds or peanuts [6,8,13,14]. In general, it has been demonstrated that autoclaving-based treatments produce a decrease in protein recovery and that this trend is more marked when treatment is accomplished under harsher conditions or when preceded by a hydration step. This phenomenon may be attributed to the numerous biochemical and structural modifications occurring on the protein moiety taking place during combined heat and pressure treatment. When combined with aggregation phenomena due to the interaction between proteins or protein-food matrix (intra- or intermolecular covalent and non-covalent interactions), this could promote protein precipitation and a consequent decrease in protein solubility and protein content in the final extract [15]. As for hazelnuts, in 2012 Lopez et al. demonstrated that autoclaving could alter the secondary and tertiary structure of tree nut proteins, inducing a glycosylation reaction [16]. In the current study, the reduced protein content observed in the treated hazelnut extracts confirms that autoclave-based treatments can modify the protein structure of hazelnut proteins, leading to a reduction in their solubility. This effect appears to be enhanced by preceding autoclaving with water incubation. In addition to reduced solubility, fragmentation of allergen proteins is likely to occur during autoclaving due to the application of pressure and temperature. It is already known that according to some Oral Food Challenge (OFC) studies [17,18] new thresholds might be proposed for allergens undergoing heating/baking treatments in food matrices, as these can induce modification of protein structure and decrease allergenicity. In line with this, it is reasonable that the reduced protein extraction or protein degradation after physical treatments might have an influence on overall SPT reactivity.

### 3.3. SDS-PAGE Profiling of Processed Hazelnuts and Protein Identifications

**Figure 4:**
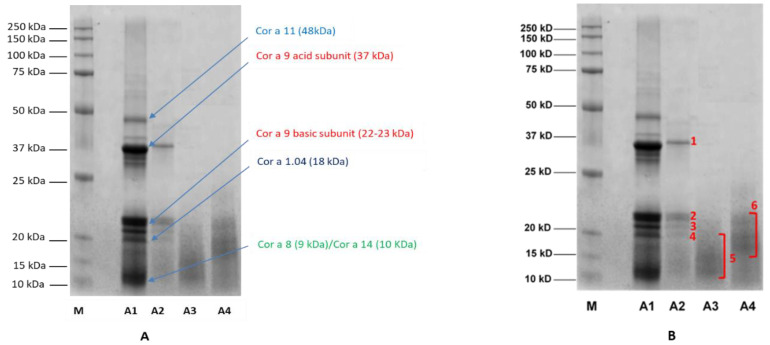
(**A**) Comparison between SDS-PAGE protein profiles of untreated (A1), autoclaved (A2), pre-hydrated and autoclaved (A3), pre-hydrated/autoclaved and subsequently dried (A4) hazelnut extract. M, MW reference standards. (**B**) Protein bands excised from the gel (numbered from 1 to 6) submitted to tryptic digestion and analysis by untargeted High Resolution Mass Spectrometry.

The raw (A1) and different processed hazelnut extracts (A2, A3 and A4) were electrophoretically profiled in order to evaluate possible changes in hazelnut protein content and structure induced by the different treatments. Specifically, 10 µg of untreated (A1) and treated hazelnut proteins (A2, A3 and A4, respectively) were analyzed by SDS-PAGE and protein profiles were observed for each sample analyzed (Figure 4). In the untreated sample (Figure 4A, lane A1), several bands in the region of 30–50 kDa and 10–22 kDa appear. As known from the literature, in the absence of reducing agents Cor a 9 (11S legumins) is organized in a hexameric structure made up of six subunits interacting non-covalently and arranged in an open ring conformation with 360 kDa [19].

Each subunit is composed by an acidic polypeptide (30–40 kDa) linked to a basic polypeptide (around 20 kDa) by a disulphide bond [20]. Under reducing conditions, acid and basic subunits are released; these are clearly visible in the lane of the untreated sample (Figure 4A, lane A1). Other bands are visible over the 37 kDa region and below 20 kDa; these are likely to be attributed to Cor a 11, Cor a 8 and Cor a 14, the MWs of which are reported to be approximately 48 Da, 9 kDa and 15–16 kDa, respectively [20]. After autoclaving (Figure 4A, lane A2), a general decrease in band intensity was recorded, with a concomitant disappearance of the protein bands at 50 kDa and below 20 kDa that were putatively attributed to the Cor a 11, Cor a 8 and Cor a 14 allergens. As for Cor a 9, a marked reduction in signals corresponding to acid and basic subunits was observed, likely attributable to reduced content of the allergen following thermal/pressure treatment. On the contrary, the protein profiles of hazelnut samples incubated with water before autoclaving (Figure 4A, lanes A3 and A4) appeared as a smear of peptides with a low MW (10–20 kDa), probably produced by fragmentation occurring during the treatments. It is worth noting that the drying process after autoclaving (A4 treatment) did not produce any significant difference in protein profile with respect to the A3 sample. 

SDS-PAGE profiles are in accordance with protein assay results (Figure 3); a progressive reduction in protein content can be seen among the treated hazelnut samples, with a more marked decrease observed for the pre-hydrated/autoclaved and pre-hydrated/autoclaved/dried samples. 

In order to obtain insight into the protein content of the specific bands identified by SDS-PAGE, the most relevant bands which were expressed differently after different treatments (Figure 4B, lanes A2, A3 and A4) were excised from the gel (the excised bands are numbered from 1 to 6), submitted to tryptic digestion, and analyzed with untargeted High Resolution Mass Spectrometry. MS spectra were then processed using Proteome Discoverer software for protein identification. Specifically, the Uniprot database referred to *Corylus avellana* (last accessed on 26 November 2021) was interrogated; the results are shown in Table 3. 

As expected, bands 1 and 2 of the autoclaved sample (Figure 4B, lane A2) were attributed to Cor a 9 allergen, suggesting the resistance of this allergen to autoclave processing (although the reduced intensity of the relative bands leads to the supposition that some partial degradation/structural alteration of the proteins likely occurred during the treatment). Cor a 9 allergen was found in bands 3 and 4 as well (Figure 4B, lane A2). The smeared bands visible in the protein profiles of the pre-hydrated/autoclaved samples, whether followed or not by drying (approximately 10–20 kDa, bands 5 and 6, respectively) (Figure 4B, lanes A3 and A4) were instead produced by a mix of peptides belonging to Cor a 9, Cor a 11 and Cor a 14 allergens (Table 3). 

The impact of autoclaving on the protein stability of tree nuts and peanuts has already been reported in the literature [6,8,16,21,22], with some papers exploring the effects of water incubation before thermal/pressure treatment [6,8]. Similar to the work described here, these authors observed that samples pretreated with water before autoclaving showed a more fragmented SDS-PAGE protein profile and were degraded in comparison with their autoclaved counterparts. To explain these phenomena, they suggested that water absorbed by seeds during incubation may facilitate the propagation of heat in the inner part of the seed, promoting protein disaggregation and a consequent decrease in band intensity. In addition, our results are in line with those described by Lopez et al. in 2012, in a study of the effects of different autoclaving and high-pressure treatment conditions on the final allergenicity of hazelnut flour. The authors highlighted the disappearance of the main hazelnut allergen protein bands in the SDS-PAGE profile of hazelnuts autoclaved at 131 °C for 15 or 20 min, ascribing these results to molecular alterations or post-translational modifications (PTMs, e.g., glycosylation) that occur during autoclaving. Indeed, by generating a series of homology-based 3D bioinformatics models, they found that the structure of the main hazelnut allergens (Cor a 8, Cor a 9) were altered after autoclaving, and found with a new glycosylation site in the Cor a 11 allergen [16]. 

### 3.4. Immunoblotting Experiments

**Figure 5:**
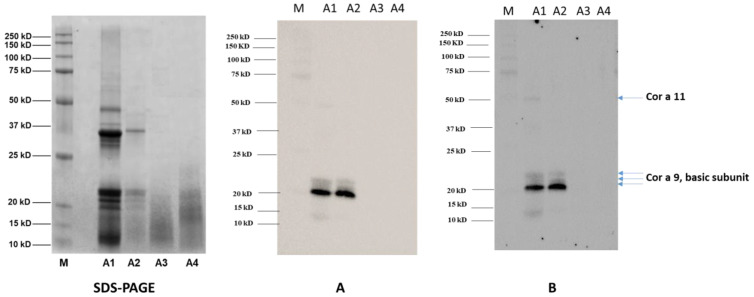

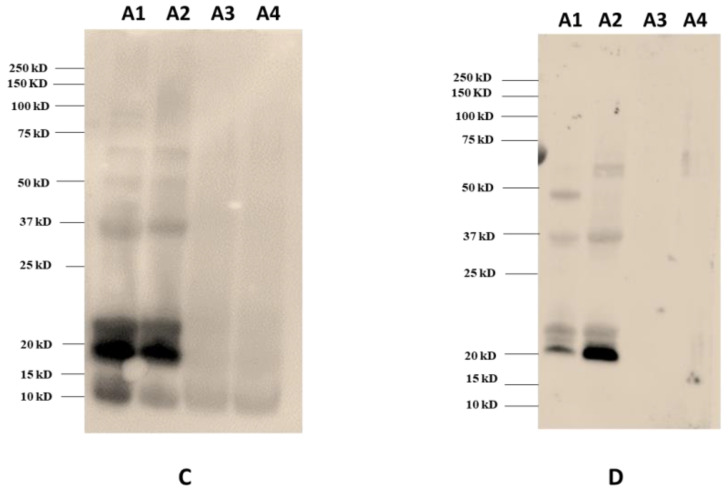
SDS-PAGE along with Immunoblots of hazelnut extract untreated (A1) or submitted to autoclaving (A2), pre-hydration/autoclaving (A3), prehydration/autoclaving/drying (A4). M, Precision Plus Protein™ All Blue Standard (10–250 kDa, Bio-Rad Laboratories, Segrate, Milan, Italy). Panel **A** and **B**: immunoblotting profiles of 14 patients allergic to hazelnuts were pooled together and incubated with goat anti-rabbit IgG Ab (Panel A) and anti-human IgE Ab (ε-chain specific) secondary antibody (Panel B). Panel **C** and **D**: immunoblotting profiles of two subgroups of 14 allergic patients (seven allergic sera pooled together for each group), both incubated with goat anti-rabbit IgG Ab.

Immunoblotting experiments were performed to assess the immunogenicity and the allergenicity of hazelnut seeds subjected to the different autoclaving treatments (A2, A3, A4). Sera from 14 patients showing different both systemic and OAS clinical symptoms were used for the immunoblotting experiments (Figure 5).

As a preliminary investigatory step, the sera of the 14 allergic patients were pooled together and used as the primary antibody, with the final aim of obtaining an overview of the effects of the autoclaving-based treatments here investigated on the final allergenicity of the hazelnut. 

The membranes were then incubated with a goat anti-rabbit IgG Ab (Figure 5A) or an anti-human IgE Ab (ε-chain specific) (Figure 5B) as the secondary antibodies. Finally, to increase the assay sensitivity two additional subgroups with seven pooled sera per group (Figure 5C,D) were incubated with a goat anti-rabbit IgG Ab (vide infra). Figure 5A shows one main reactive band with MW of approximately 20 kDa in untreated hazelnut (lane A1), along with weak intensity bands at 50 kDa and in the range of 10 and 15 kDa. Two additional blurred signals between 21–22 kDa are displayed in the A1 sample as well. According to the literature, protein banding at 20 kDa can be putatively attributed to the basic subunit Cor a 9 hazelnut allergens, as can bands in the range of 21–22 kDa. On the contrary, the reactivity displayed at 50 kDa could be ascribable to the Cor a 11 allergen, which has an original MW of 48–50 kDa, while the weak signal in the range of 10–15 kDa could be attributed to Cor a 8 or Cor a 14 allergens, which have MWs falling in this range. After autoclaving treatment (Figure 5A, lane A2), the weak reactivity of the signals at 50 kDa and below 15 kDa became negligible, while the intense spot at 20 kDa and bands in the range of 21–22 kDa persisted. On the basis of proteomic investigation accomplished with SDS-PAGE protein profiles, these bands can be attributed to Cor a 9 and its isoform allergen Cor a 9.0101 (Table 3), confirming that these allergens survived the autoclaving processing and preserving their allergenic potential. On the contrary, no reactive signal was displayed for the band at 50 kDa, putatively ascribed to Cor a 11, or for the 10–15 kDa hazelnut allergens (likely Cor a 8 and Cor a 14), confirming the susceptibility of these proteins to autoclave processing (Figure 5A). As for the pre-hydrated/autoclaved (Figure 5A, lane A3) and pre-hydrated/autoclaved/dried (Figure 5A, lane A4) samples, no clearly identifiable reactive bands were shown, thus confirming the key role of the water imbibition step in the structural and conformational alteration/degradation phenomena induced and enhanced by the thermal/pressure treatment applied on these proteins. Cor a 9 was demonstrated to be a very well-structured protein, enriched with a beta-sheet core and containing long unstructured loops. These loop regions were found to be structurally unstable, and were predicted to retain linear epitopes located at the external faces of the protein and thus exposed to solvent [16]. After submitting hazelnut samples to autoclaving (121 °C or 138 °C for 15 or 30 min), Lopez and co-workers obtained a reduction of Cor a 9 allergenicity, with no bands corresponding to this protein visible in the SDS-PAGE analysis. In light of this, the authors hypothesized that the allergenicity of Cor a 9 could be predominantly ascribed to structural conformation and not to linear epitopes [16]. Our results seem to support this hypothesis. Indeed, the enhanced action of previous water incubation on autoclaving (A3, A4) seems effective in impairing the structure of Cor a 9, with no reactive epitopes surviving after processing, which is different from the results for the solely autoclaved sample (Figure 5A, lane A2). On visual inspection of the SDS-PAGE (pictured in Figure 4B), despite the visible smear bands below 20 kDa produced by a mixture of Cor a 9 and Cor a 14 allergens peptides in the A3 sample and by Cor a 9, Cor a 14 and Cor a 11 allergens peptides in the A4 sample (as highlighted by proteomic investigation in Table 3), no reactive signals were displayed in the putatively corresponding lanes of the immunoblotting profiles. As already indicated by the electrophoretic analysis, no significant changes in immunoblotting profile or in the consequent final allergenicity of pre-hydrated/autoclaved hazelnut were found by drying the sample (Figure 5, lane A3). 

Similar results were obtained by incubating the A1-A4 samples with anti-human IgE Ab (vide infra) (ε-chain specific) secondary antibody, confirming that a specific IgE can bind protein bands detected in untreated and autoclaved samples, and thus likely trigger an allergic reaction in vivo. On the contrary, no IgE reactivity was displayed for the A3-A4 samples, indicating their likely lack of allergic potential.

Considering that sensitization to specific hazelnut proteins could vary among allergic individuals, additional immunoblotting experiments were performed by dividing the 14 patient sera into two groups of seven patient sera. The aim was to unveil possible different patterns of sensitization in different patient subgroups. In particular, Figure 5C,D shows immunoblotting experiments conducted using two different serum pools, each of which was made up of seven patients’ sera. These immunoblots showed different sensitivities compared to those shown in Figure 5A,B, which used a pool made up of all 14 patients’ sera, depending on the differential antibody concentration of each serum sample in the subgroups.

As a matter of the fact, the reactivity profile varied among the different pools. Figure 5C shows more intense bands at approximately 50 kDa (Cor a 11), 37 kDa (Cor a 9 acid subunit), 20–22 kDa (Cor a 9 basic subunit/Cor a 1.04) and 10 kDa (Cor a 8/Cor a 14) when compared to Figure 5A,B,D. This was expected, as pools represent patients’ average response and can therefore vary along with the differential pool array. Further analyses of individual patient sera are necessary in order to accurately identify the individual reactivity profile and how it could be modified by specific treatments. This study is a preliminary analysis primarily focused on the general effectiveness of the physical treatment used to reduce allergenicity. A pool of sera containing different IgE antibodies specificities was considered useful in answering this research question; however, the findings provided here will be expanded upon in subsequent studies in which immunoblot analyses will be performed on individual patients in parallel with specific IgE profiles based on component-resolved diagnosis. Using this approach together with Mass Spectrometry analysis of protein fragments should permit better understanding of which specific allergenic proteins are more liable to degradation, and consequently which specific IgE reactivities will be lost. This will enable prediction of individual patients’ absence of reactivity to modified hazelnut (i.e., no reaction after exposure to modified hazelnut) by analyzing individual IgE profiles against each antigenic protein obtained by component-resolved diagnosis.

The effect of the autoclaving on the final allergenicity of hazelnuts was already investigated in 2012 by Lopez et al. The authors submitted hazelnut defatted flour to autoclave processing under different conditions (121 °C 15 min, 121 °C 30 min, 138 °C 15 min, 138 °C 30 min) and investigated the IgE-reactivity of fifteen allergic patients via Western blot experiments. In addition, they studied the changes to the conformational structure of hazelnut allergens induced by autoclaving by generating a series of homology-based 3D bioinformatics models for the allergens Cor a 1, Cor a 8, Cor a 9, and Cora 11. As result, the authors observed that under harsher conditions (138 °C 15 min, 138 °C 30 min) autoclaving induced a severe reduction in hazelnut allergenicity in the patients studied. Indeed, the specific IgE binding of certain immunoreactive hazelnut protein bands such as Cor a 1, Cor a 8, Cor a 9 and (vide infra) Cor a 11 all decreased. Moreover, the structural analysis (3D modelling) of these allergens highlighted that, vide infra, relevant glycosylation occurred in the protein allergen Cora 11 after autoclaving, suggesting that the combination of temperature and pressure could promote the interaction of protein and matrix, likely altering the final allergenicity of the protein [16].

Very recently, Cuadraro et al. investigated the effects of autoclaving on the final allergenicity of the Cor a 9, Cor a 14 and Cor a 8 hazelnut allergens by testing two different autoclaving temperatures, 121 °C and 138 °C, for 30 min. Whole hazelnut seeds were processed and, similar to the results described by Lopez et al., they observed in the different immunoblot profiles a marked reduction of Cor a 9, Cor a 14, and Cor a 8 reactivity after autoclaving hazelnut material at 138 °C for 30 min [23].

In the present investigation, we observed that by autoclaving hazelnut seeds at 134 °C for 10 min the intensity of Cor a 9 band appeared to be reduced, with the disappearance of the 50 kDa and 10 kDa bands putatively ascribed to the Cor a 11 and Cor a 8 allergens (as shown in the SDS-PAGE picture in Figure 3, lane A2). These results are comparable to those obtained by the aforementioned authors when applying similar autoclaving conditions, that is, a temperature of 121 °C for 30 min [16,23].

Furthermore, the immunoblotting profiles presented here confirm the IgG and IgE reactivity of Cor a 9 basic subunits after the autoclaving of hazelnut material only (Figure 5A–D). On the contrary, by incubating hazelnut seeds with water before autoclaving the full degradation/fragmentation of the proteins was observed, with the disappearance of the main allergenic bands in both the corresponding SDS-PAGE (Figure 3, lane A3 and A4) and the immunoblotting profile (Figure 5A–D). These results are comparable to those observed by Lopez et al. and Cuadraro et al. when analyzing hazelnut autoclaved at 138 °C for 30 min via SDS-PAGE and Western blotting [16,23]. In the light of this, it is reasonable to suppose that water incubation strengthens the alterative phenomena induced by autoclaving on proteins, obtaining the same effects as autoclaving of hazelnut under such harsh conditions as 138 °C for 30 min. 

## 4. Conclusions

This preliminary study shows that autoclaving treatment appears to be effective in reducing the allergenicity of hazelnut proteins in most of the patients herein screened by skin prick test, especially when preceded by hydration and/or followed by drying, probably due to denaturation/fragmentation of most of the major and minor allergenic proteins. The reduction in hazelnut protein solubility due to the specific treatment applied must be considered when interpreting the reduced allergenicity revealed by immunoblot tests. Taking into account that the immunoblotting experiments were accomplished by loading the same amount of proteins for all treated samples, the reduction of reactivity in the observed bands can be reasonably attributed to the reduced allergenicity of those proteins. The appearance of a smear corresponding to low-MW proteins (lower than 20 kDa) along with the absence of higher MW bands suggests possible protein fragmentation caused by physical treatments, which could be responsible for reduced IgG and IgE reactivity. Furthermore, unique peptides corresponding to Cor a 9 and Cor a 11 were detected in the low-MW degraded proteins (protein bands 5 and 6 in Figure 4B and Table 3), confirming the hypothesis of protein degradation. Notably, Cor a 9 and Cor a 11 are two of the three most important and harmful hazelnut allergens that cause systemic reactions. The third allergen causing systemic reactions, Cor a 8, was likely present in the band at approximately 10 kDa. However, possible fragmentation of this protein can only be hypothesized, not demonstrated, as the fragments resulting from the effects of each treatment were be too small to be identified with our immunoblots and could have escaped the gel meshes during gel running. Further specific experiments must be performed in order to confirm this hypothesis. Nonetheless, according to studies based on oral food challenges there are threshold doses below which allergic reactions do not occur. Consequently, it is possible that the reduced protein extraction after physical treatments could have influenced SPT reactivity. Whether the reduced SPT reactivity that we observed in our patients might be correlated to a decreased response to OFC needs to be elucidated, and will be investigated in further studies.

In conclusion, based on both patients’ reactivity in vivo (SPT) and their immunoblotting profiles, this study showed that specific physical treatments can reduce the allergenicity of hazelnuts. These results need to be confirmed via double blind placebo-controlled food challenge with the treated extracts. Moreover, further analysis aimed at investigating the reactivity of treated hazelnuts in individual patients, combined with deep analysis of their IgE profiles based on component-resolved diagnosis, will enable prediction of individual patients’ non-reactivity to modified hazelnut (i.e., no reaction after exposure to treated hazelnut) by analyzing patient IgE profiles against each individual hazelnut antigenic protein obtained by component-resolved diagnosis.

Finally, from the perspective of using these physical treatments in food production, their effects on the organoleptic properties of the preparation (i.e., smell, taste) is worthy of further investigation in order to optimize the process for commercial purposes.

## Figures and Tables

**Table 1:** Hazelnut allergens: overview of different protein families, MWs and specific related clinical pictures.

Protein Name	Protein Family	MW (kDa)	Clinical Picture
Cor a 1.04	PR-10 (Bet v 1 like)	17	OAS
Cor a 2	profillin (Bet v 2 like)	14	OAS
Cor a 8	LTP ^1^	9	Systemic reactions
Cor a 9	SSP ^2^ (11S globulin-legumin like)	40	Systemic reactions
Cor a 11	SSP (7S globulin-vicilin like)	48	Systemic reactions
Cor a 12	Oleosin	17	
Cor a 13	Oleosin	14–16	
Cor a 14	SSP (2S albumin)	10	

^1^ LTP = lipid transfer protein; ^2^ SSP = seed storage protein.

**Table 2:** SPT reactivity of patients to the different treated hazelnut extracts.

Extract	Reactive Patients N (%)	Wheal Area, mm^2^ Median (IQR)	Range, mm^2^	*P*
A1	22 (100%)	22.7 (16.9, 29.5)	9–67	Reference
A2	6 (27.3)	0 (0–10.5)	0–21	*p* < 0.01 ^2^
A3	2 (9.1)	13.5 (patient #15) 4.5 (patient #20)	-	n.e. ^1^
A4	1 (4.5)	13 (patient #15)	-	n.e. ^1^

^1^ n.e. = not estimable. ^2^ median and interquartile range (IQR) were calculated considering all 22 data points; 16 out of 22 patients did not show any reactivity. Reference = this group (A1) was used for comparisons with the other groups (A2, A3, A4) for statistical analyses. # = number.

**Table 3:** Summary of proteins identified by Proteome Discoverer software referred to SDS-PAGE protein bands excised and in-gel digested from protein pattern of autoclaved (A2), prehydrated/autoclaved (A3), and prehydrated/autoclaved/dried (A4) hazelnut samples.

Sample	Band	Accession	Description	Allergen	Coverage (%)	#Peptides (Unique)	Score
A2	1	A0A0A0P7E3	Cor a 9 allergen (*Corylus avellana*)	Cor a 9	52	21 (2)	124
		Q8W1C2	11S globulin-like protein (*Corylus avellana*)	Cor a 9.0101	45	23 (2)	112
	2	A0A0A0P7E3	Cor a 9 allergen (*Corylus avellana*)	Cor a 9	44	17 (4)	207
		Q8W1C2	11S globulin-like protein (*Corylus avellana*)	Cor a 9.0101	37	14 (1)	202
	3	A0A0A0P7E3	Cor a 9 allergen (*Corylus avellana*)	Cor a 9	40	16 (3)	118
		Q8W1C2	11S globulin-like protein (*Corylus avellana*)	Cor a 9.0101	33	14 (1)	116
		A0A1I9RG40	Ribulose bisphosphate carboxylase large chain (*Corylus avellana*)		19	8 (8)	16
	4	A0A0A0P7E3	Cor a 9 allergen (*Corylus avellana*)	Cor a 9	28	11 (11)	38
A3	5	Q8W1C2	11S globulin-like protein (*Corylus avellana*)	Cor a 9.0101	32	18 (2)	111
		A0A0A0P7E3	Cor a 9 allergen (*Corylus avellana*)	Cor a 9	34	19 (2)	109
		D0PWG2	2S albumin (*Corylus avellana*)	Cor a 14/Cor a 14.0101	44	8 (8)	15
A4	6	A0A0A0P7E3	Cor a 9 allergen (*Corylus avellana*)	Cor a 9	30	14 (2)	98
		Q8W1C2	11S globulin-like protein (*Corylus avellana*)	Cor a 9.0101	28	14 (2)	98
		D0PWG2	2S albumin (*Corylus avellana*)	Cor a 14/Cor a 14.0101	31	8 (8)	4
		Q8S4P9	Vicilin Cor a 11.0101 (*Corylus avellana*)	Cor a 11/Cor a 11.0101	8	2 (2)	2

# = number.
